# *In silico* Mechano-Chemical Model of Bone Healing for the Regeneration of Critical Defects: The Effect of BMP-2

**DOI:** 10.1371/journal.pone.0127722

**Published:** 2015-06-04

**Authors:** Frederico O. Ribeiro, María José Gómez-Benito, João Folgado, Paulo R. Fernandes, José Manuel García-Aznar

**Affiliations:** 1 IDMEC, Instituto Superior Técnico, Universidade de Lisboa, Lisbon, Portugal; 2 Multiscale in Mechanical and Biological Engineering (M2BE), Aragón Institute of Engineering Research (I3A), University of Zaragoza, Zaragoza, Spain; Institute for Frontier Medical Sciences, Kyoto University, JAPAN

## Abstract

The healing of bone defects is a challenge for both tissue engineering and modern orthopaedics. This problem has been addressed through the study of scaffold constructs combined with mechanoregulatory theories, disregarding the influence of chemical factors and their respective delivery devices. Of the chemical factors involved in the bone healing process, bone morphogenetic protein-2 (BMP-2) has been identified as one of the most powerful osteoinductive proteins. The aim of this work is to develop and validate a mechano-chemical regulatory model to study the effect of BMP-2 on the healing of large bone defects *in silico*. We first collected a range of quantitative experimental data from the literature concerning the effects of BMP-2 on cellular activity, specifically proliferation, migration, differentiation, maturation and extracellular matrix production. These data were then used to define a model governed by mechano-chemical stimuli to simulate the healing of large bone defects under the following conditions: natural healing, an empty hydrogel implanted in the defect and a hydrogel soaked with BMP-2 implanted in the defect. For the latter condition, successful defect healing was predicted, in agreement with previous *in vivo* experiments. Further *in vivo* comparisons showed the potential of the model, which accurately predicted bone tissue formation during healing, bone tissue distribution across the defect and the quantity of bone inside the defect. The proposed mechano-chemical model also estimated the effect of BMP-2 on cells and the evolution of healing in large bone defects. This novel *in silico* tool provides valuable insight for bone tissue regeneration strategies.

## Introduction

The repair of critical size bone fractures is an important challenge facing modern orthopedics researchers. The gold standard procedure for critical size defect repair consists of filling the defect with autografted bone harvested from the iliac crest [1]. However, in addition to the limited availability of graft tissue, this practice is associated with limitations such as poor vascularisation and poor bone remodeling, which can compromise full recovery [2]. To overcome these problems, the development of new methods to promote bone regeneration in critical size fractures has been an important focus in tissue engineering [3–9].

In 1965, Marshall Urist demonstrated that the organic component of bone induces bone formation and that this bone morphogenetic potential was due to one or more proteins [10]. Those bone morphogenetic proteins (BMPs) were purified and sequenced in the late 1980s [11,12], and they are now produced *via* recombinant DNA technology (rhBMP) [12,13].

Of the 20 proteins that compose the BMP family, BMP-2 appears to be the most relevant osteoinductive growth factor [14,15] for bone formation [16,17] and bone healing [18,19]. BMP-2 promotes cell chemotaxis, proliferation and differentiation towards the osteogenic pathway as well as extracellular matrix production, protein secretion and mineralization [20]. Futhermore, the beneficial effects of BMP-2 on fracture healing are well established [21], and medical devices releasing BMP-2 have already been designed and approved by the FDA (Food and Drug Administration) [22,23], specifically for spinal fusions and open tibia fractures.

Several computational mechanoregulatory models have been proposed to study natural bone fracture healing [24–27]. Claes et al. [28] computed stress and strain in sheep tarsal fractures and determined a set of rules for tissue differentiation. Lacroix and Prendergast [29], following the differentiation rule proposed by Prendergast et al. [30], presented a biomechanical model where tissue differentiation; within a fixed callus geometry; is stimulated by fluid flow and shear strain. In the work of Gómez-Benito et al. [31] cell populations *per se* are introduced, cellular differentiation is influenced by the local mechanical environment and callus geometry growth is also simulated. Despite the primary focus being mechanical stimuli alone, Bailón-Plaza and van der Meulen [32] considered chondrogenic and osteogenic growth factor gradients to drive tissue differentiation and bone healing. Additionally, the latter bioregulation model was complemented with a mechanical stimulus [33] and angiogenesis [34,35] within a mechanobioregulatory framework.

In the view of bone tissue regeneration, computational models have been applied to study bone growth inside scaffolds. Adachi et al. [36] first developed a computational framework to study the balance between scaffold degradation and new bone formation. Using a mechanoregulatory algorithm, Kelly and Prendergast [37] proposed ideal mechanical properties in a scaffold for cartilage regeneration in osteochondral defects. Similarly, Byrne et al. [38] addressed the necessary commitment between scaffold properties such as porosity, elasticity and scaffold degradation to improve bone regeneration by combining a mechanoregulation algorithm with a 3D lattice approach. Sanz-Herrera et al. [39] considered a mathematical phenomenological model to evaluate bone regeneration inside a scaffold and qualitatively compared it with previous *in vivo* experiments [40]. Recently, Checa and Prendergast [41] studied the effects of cell seeding and mechanical loading inside a biodegradable scaffold on scaffold vascularisation and tissue ingrowth.

More recently, *in silico* models have been developed in order to provide a better understanding of large bone union/impaired healing. So, Carlier et al. [42,43] developed a multi-scale approach to study the influence of vascularization during bone healing. Importantly, oxygen was shown to be a critical element for a successful healing to be attained in large bone defects. In addition, Moore et al. [44] also developed an *in silico* model to study the “one stage transport” surgical procedure which considers a periosteum layer surrounding the bone defect. In this model, BMP-2 production increased with higher levels of strain in the periosteum. The endogenous increase of BMP-2 levels increased cell activity and bone tissue production, and bone bridging in large bone defects was successfully predicted.

To the best of our knowledge, we did not find any previous computational studies assessing the use of growth factor delivery devices in large bone defects, due to the very fact that mechanical and chemical stimuli are traditionally treated separately. In order to tackle this issue, we hypothesize that the mechanical stimulus coordinated by the chemical stimulus can provide an integrated outline to deal with systems where a combination of both stimuli plays an important role. Thus, we propose in this work an *in silico* mechano-chemical model with the purpose of predicting the healing in large bone defects, given the effect of BMP-2 released in the defect. Given this, we present the following results: (1) based on the argument that BMP-2 is one of the most important growth factors involved in bone fracture healing [21,45–47], we collected quantitative data on the effects of BMP-2 on cell proliferation, migration, differentiation, maturation and extracellular matrix (ECM) production (2) the effects of BMP-2 on cells were then incorporated into a mechanoregulatory model, hence combining and integrating mechanical and biological stimuli [48] and (3) finally, we defined a mechano-chemical model to simulate healing of a critical size fracture with a hydrogel incorporated as a BMP-2 delivering device. The results obtained were then compared with the *in vivo* results of Boerkel et al. [49] on the healing of large femoral defects in mice.

## Materials and Methods

To incorporate BMP-2 into mechanobiological healing bone models, we searched for experimental data describing the effect of BMP-2 on cellular behavior. Additionally, we described the equilibrium between BMP-2 production and consumption. Then, we developed a model to simulate an alginate hydrogel used to deliver BMP-2, as used in the *in vivo* study by Boerckel et al. [49]. We evaluated *in silico* the healing potential of BMP-2 in a rat femoral large bone defect.

### Effect of BMP-2 on cells

Many aspects of cell behavior are influenced by BMP-2, including chemotaxis, proliferation, differentiation towards the osteogenic pathway, ECM production and mineralisation [20]. However, the effect of BMP-2 on cell activity is dose-dependent [49–51]. Therefore, in our approach we considered BMP-2 dose-dependent chemical stimulus, represented by *g* (in *ng*. *cm* ^−3^). Next, we focused on the effect of BMP-2 for each of the enumerated aspects of cellular activity.

**Fig 1:**
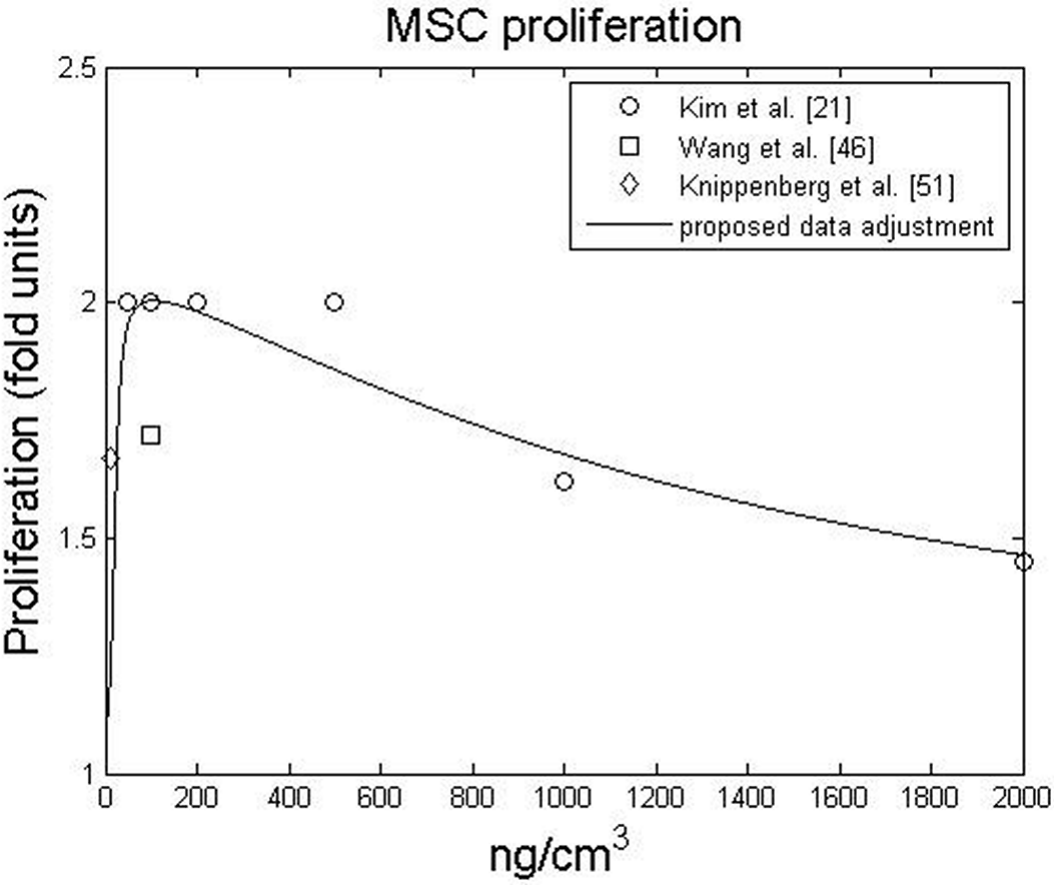
Influence of BMP-2 concentration on MSC proliferation. Proliferation is expressed as a fold increase relative to proliferation without BMP-2

Cell proliferation is an increase in the cell population due to cell division. In general, BMP-2 regulates cell proliferation [47,52,53] in a dose-dependent manner. Knippenberg et al. [52] observed that the number of cells nearly doubled when cells were treated with BMP-2 at a concentration of 10 *ng*. *cm* ^−3^ was added. However, Kim et al. [21] observed that cell proliferation was almost unchanged for BMP-2 concentrations between 50 and 200 *ng*. *cm* ^−3^. Moreover, for high concentrations of BMP-2 ranging from 500 – 2000 *ng*. *cm* ^−3^, cellular proliferation was significantly reduced in a dose-dependent manner. These observations are summarised in Fig 1, which illustrates the impact of BMP-2 on cell proliferation. In the proposed data adjustment, medium without BMP-2 does not exert an influence on cell proliferation, there is a strong effect on proliferation for low concentrations of BMP-2, a threshold of cellular proliferation is reached within the 50 – 200 *ng*. *cm* ^−3^ concentration range and proliferation is reduced at higher concentrations of BMP-2. It should be noted that the fold values are relative to a control experiment lacking BMP-2, performed by the referenced authors.

**Fig 2:**
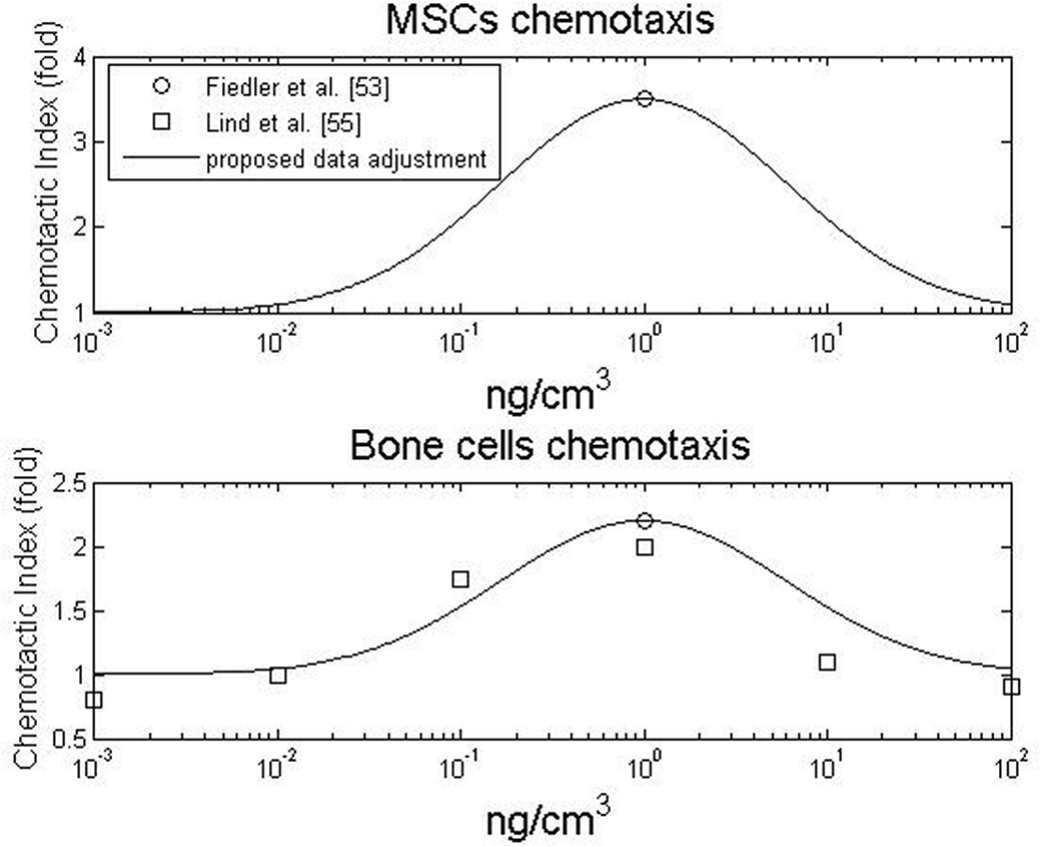
Influence of BMP-2 concentration on MSC (top) and bone cells (bottom) chemotaxis. Chemotaxis is expressed as a fold increase relative to cell migration without BMP-2

Cell migration is also influenced by BMP-2, as BMPs exert a chemotactic effect on cells (i.e., cellular attraction to a chemical) [54,55]. Lind et al. [56] observed that cellular chemotaxis is a BMP-2-dependent bell-shaped function centered on a BMP-2 concentration of 1 *ng*. *cm* ^−3^. There is *grosso modo* an increase in the chemotactic effect of BMP-2 from 0.01 *ng*. *cm* ^−3^ to 1 *ng*. *cm* ^−3^ followed by a decrease for concentrations from 1 *ng*. *cm* ^−3^ to 100 *ng*. *cm* ^−3^ [56]. Lind et al. [56] also found that the peak at 1 *ng*. *cm* ^−3^ promoted a 2.2-fold increase in osteoblast chemotaxis, and similar results were observed for U2-OS cells (primitive transformed cells of mesenchymal origin). Fiedler et al. [54] observed that 1 *ng*. *cm* ^−3^ of BMP-2 stimulated 2.2-fold and 3.5-fold increases in the migration of osteoblasts and mesenchymal progenitor cells (or MSCs) respectively. Fig 2 summarizes the dose-dependent, chemotactic effect of BMP-2 on MSCs and osteoblasts.

During fracture healing, MSCs can differentiate into cartilage cells, fibroblasts and bone cells [57]. BMP-2 is essential for bone healing [18] and is present in the fracture gap throughout healing [18,58]. However, some studies have concluded that BMP-2 inhibits the cartilage phenotype [52], which is essential for soft callus formation [45] and subsequent endochondral ossification [59]. Moreover, when cells go through a certain differentiation path, this is usually measured by gene expression which does not allow an objective quantification of the fold increase of cell differentiation due to BMP-2. Consequently, in order to have a well-defined set of differentiation rules, we followed a mechanistic approach [60], which states that the mechanical deformation provides enough stimulus for cells to differentiate [61,62]. So far this approach could be the classical one taken by many authors in the topic of bone healing simulation [24–29]. Nonetheless, BMP-2 is essential to trigger healing [45,46] and for that reason we assume that at least a minimal physiological concentration (*g*_*min*_) is required for cellular activity to occur.

**Fig 3:**
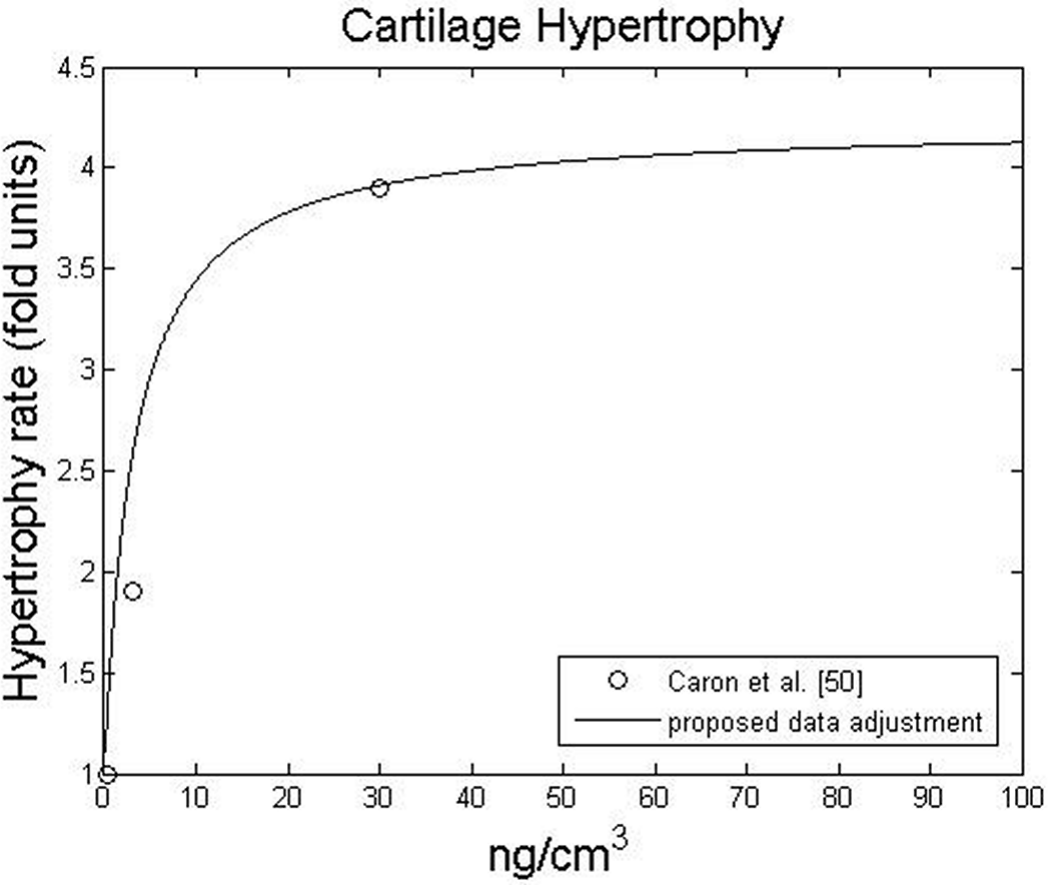
Influence of BMP-2 on chondrocyte hypertrophy. Chondrocyte hypertrophy is expressed as a fold increase relative to chondrocyte hypertrophy without BMP-2.

In recent years, the effect of BMP on cartilage hypertrophy has been studied [51,63]. Notably, Caron et al. [63] demonstrated that BMP-2 had almost no influence on the hypertrophic phenotype at concentrations below 0.3 *ng*. *cm* ^−3^. However, chondrocyte maturation is dose-dependent for higher concentrations of BMP-2. For example, cartilage hypertrophy increased approximately 2-fold at a concentration of 3 *ng*. *cm* ^−3^ and approximately 3.8-fold at a concentration of 30 *ng*. *cm* ^−3^ [63]. The effect of BMP-2 on chondrocyte maturation is summarised in Fig 3. We were not able to find data on the effect of very high BMP-2 concentrations on chondrocyte hypertrophy. Thus we assumed an asymptotic behaviour at 3.8-fold for concentrations above 30 *ng*. *cm* ^−3^.

**Fig 4:**
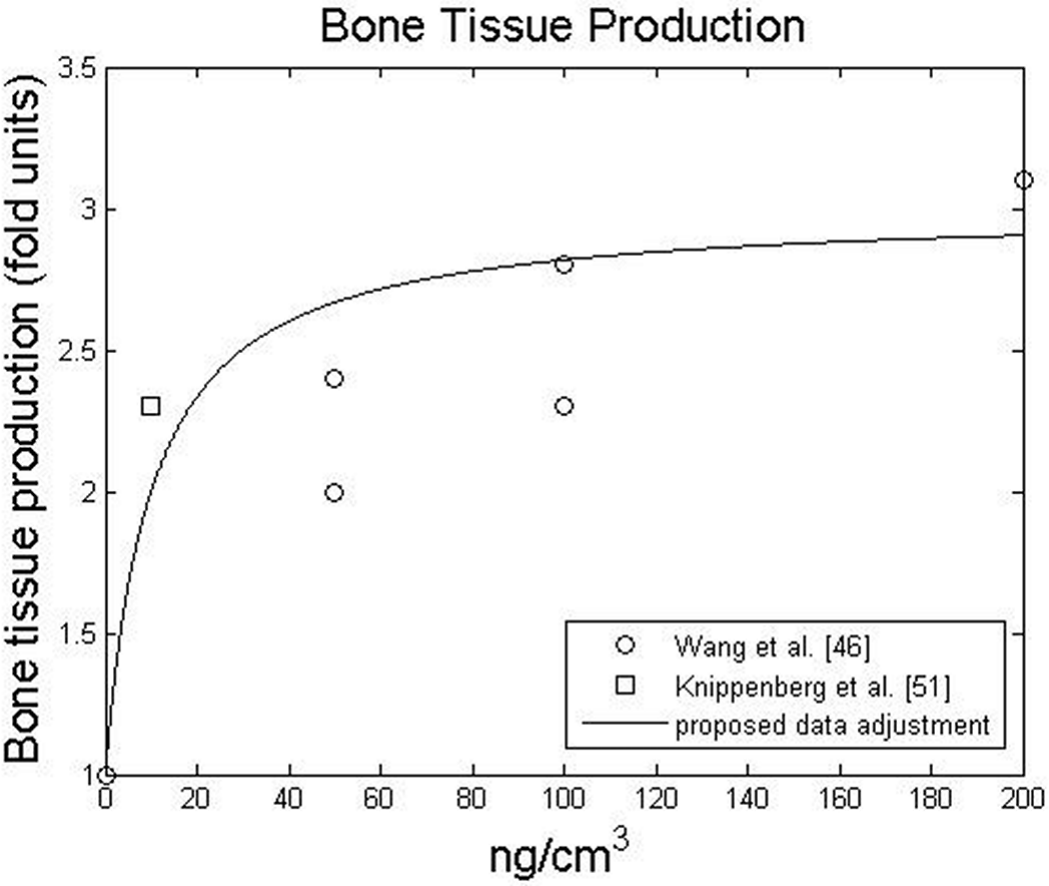
Influence of BMP-2 on bone tissue production. Bone tissue production is expressed as a fold increase relative to bone tissue production without BMP-2

After the experiments performed by Urist [10], BMPs have been increasingly researched for their effects on bone cells and tissue. Recently, the osteoinductive power of BMPs has been stressed by many authors [45,47,52,64–67]. In many studies, the osteoinductive potential of BMP-2 has been explored not by measuring the number of bone cells *per se*, but rather by the amount of extracellular proteins produced [45,66] and their relative gene expression levels [47,52]. From a proteomic perspective, Ryoo et al. [66] and Tsuji et al. [45] noticed increases in osterix and osteocalcin production in the presence of BMP-2. From a gene expression perspective, Knippenberg et al. [52] observed that 10 *ng*. *cm* ^−3^ of BMP-2 promoted a 2.3-fold increase in osteopontin expression. More recently, Wang et al. [47] observed dose-dependent proportional increases in the expression of osteocalcin (up to 3.1-fold) and osterix (up to 5.5-fold) within the 0 – 200 *ng*. *cm* ^−3^ concentration range. Based on these data and assuming the existence of an asymptote at higher concentration [67], the effect of BMP-2 on bone tissue production is presented in Fig 4.

Thus far, we have quantitatively described how cells are affected by BMP-2 supplemented medium. However, to understand the effect of BMP-2 at normal physiological concentration, we must first determine the physiological concentration range. A wide range of values was found in the literature. For example, Huang et al. [68] studied the amounts of BMP-2 produced by osteoprogenitors and osteoblasts *in vitro* and found concentrations of 0.01 – 0.3 *ng*. *cm* ^−3^. Similar values have been reported in other studies, including those of Park et al. [69] and Wang et al. [70], who measured average concentrations of 0.087 *ng*. *cm* ^−3^ and 0.072 *ng*. *cm* ^−3^ respectively, in healthy human subjects. However, higher values have also been reported. For example, Wang et al. [71], found their control group to have an average concentration of 0.51 *ng*. *cm* ^−3^. Based on this data, we can say that BMP-2 physiological concentration can be globally found within the 0.008 – 0.5 *ng*. *cm* ^−3^ range. Consequently, at normal physiological conditions, the cellular processes of proliferation, chemotaxis, maturation and matrix production are very close to 1-fold.

### 2.2. Modelling the dynamics of BMP-2

Data on BMP-2 can be used to modulate cellular behavior. To define the equilibrium equation of BMP-2, we considered that BMP-2 concentration, *g*, can vary due to cellular consumption (subdivided between background consumption, and consumption due to cellular events [68]), molecular half-life degradation, cellular production and molecular diffusion:

$$\frac{\partial g}{\partial t}=-\left(\frac{\partial g_{events}}{\partial t}+\frac{\partial g_{background}}{\partial t}\right)-\frac{\partial g_{degradation}}{\partial t}+\frac{\partial g_{production}}{\partial t}+\frac{\partial g_{diffusion}}{\partial t}$$ (1)

Cell proliferation, cartilage cell hypertrophy, bone production and cell differentiation can all be considered liable cell events, capable of causing variations in BMP-2 concentration. Because proliferation increases the number of cells, we assumed that physiological levels of BMP-2 would be replenished quickly enough. However, we underestimated BMP-2 consumption due to cartilage hypertrophy as well as bone tissue production. Therefore, BMP-2 consumption due to differentiation alone stands as

$$\frac{\partial g_{events}}{\partial t}=\delta_{differentiation}.\left(g-g_{min}\right)$$ (2)

where *δ*_*differentiation*_ is an indicator function (*δ* = 1 if there is differentiation, *δ* = 0 otherwise), *g* is the concentration of BMP-2 and *g*_*min*_ is the low boundary of physiological concentration. As described by Eq (2), whenever a differentiation event occurs, such as MSC differentiation towards chondroblastic or osteoblastic fates, all the BMP-2 in the medium is consumed, resulting in the minimum physiological BMP-2 level: *g*_*min*_ = 0.008 *ng*. *cm* ^−3^ [68]. BMP-2 was assumed to have no effect, on the fibroblastic differentiation pathway.

As stated by Lander et al. [72] and Umulis et al. [73], BMP-2 molecules in an aqueous medium bind reversibly with BMP-2 receptors (BMPR). When bound, the BMP-BMPR complex can undergo endocytosis. The kinetics of this process are analogous to Michaelis-Menton kinetics [73] considering BMP-2 as the substrate and BMPR as the catalyst, such that:

$$\frac{\partial g_{background}}{\partial t}=\frac{V_{max}\;g}{K_{m}^{a}+g}$$ (3)

where $K_{m}^{a}$ is analogous to the Michaelis constant and *V*_*max*_ is the maximum rate at which BMP-2 is consumed [73]. MSCs and bone cells are assumed to be the main contributors to BMP-2 consumption [20,45,47,68,74].

*In vivo* BMP-2 half-life was also considered to take part in molecule degradation and was taken into account as shown in Eq (1). We propose that the degradation behavior follows an exponential decay with a half-life *t*_1/2_ = 0.42 *days* as determined by Bramono et al. [75]:

$$\frac{\partial g_{degradation}}{\partial t}=ge^{-\lambda_{1/2}^{in\;vivo}t}$$ (4)

where the $\lambda_{1/2}^{in\;vivo}$ is the half-life rate parameter such as $\lambda_{1/2}^{in\;vivo}=\frac{\text{ln}\;2}{t_{1/2}}$.

We define BMP-2 production term due to cell activity as

$$\frac{\partial g_{production}}{\partial t}=\alpha_{prod}\frac{\left(c_{s}+c_{b}\right)}{\gamma g+\gamma_{0}}$$ (5)

where *c*_*s*_ and *c*_*b*_ are the MSC and bone cell concentrations respectively, and *α*_*prod*_, *γ* and *γ*_0_ are three parameters. These three parameters are adjusted to guarantee the following: (1) cells require the minimum BMP-2 concentration in their medium, thereupon they will secrete it more quickly if the levels are sub-physiological and (2) when the BMP-2 level is within physiological range (0.008 – 0.05 *ng*. *cm* ^−3^), the production rate must not drive *g* outside of that range. Note that the proposed physiological range comprises the endogenous BMP-2 concentration observed during murine fracture healing range (18 – 22 *pg*/*ml*) [76].

Together, the background consumption and the production rates promote a rapid input of BMP-2 when its levels are low as well as a faster consumption when it is in excess, hence guaranteeing the maintenance of normal physiological levels.

Finally, we defined BMP-2 diffusion according to Fick’s Law

$$\frac{\partial g_{diffusion}}{\partial t}=D_{g}\nabla^{2}g$$ (6)

where *D*_*g*_ is the diffusion coefficient of BMP-2.

### 2.3. Alginate hydrogel

Alginate hydrogels are biocompatible and stable colloidal networks [77] useful in bioengineering applications such as tissue engineering and drug delivery devices [50,78,79]. According to Boerckel et al. [49], the use of an alginate hydrogel serves two main purposes: 1) to control the BMP-2 release profile and 2) to provide support for cell migration. Moreover, in our model we also considered degradation kinetics.

In the literature, several authors have provided a detailed description of the release [80,81], degradation [82] and swelling [83] mechanisms of hydrogels in the light of thermodynamics theory. Nevertheless, the system approached here is complex and besides the release, degradation and swelling mechanisms, we also have to consider cells and cell-hydrogel interactions such as cell migration and enhanced hydrolysis due to cellular influence. Therefore, we decided to use a simpler approach as described below.

Concerning BMP-2 release, the release profile of BMP-2 from an alginate hydrogel presents a good fit with an exponential decay, as shown by Boerckel et al. [49]. We hypothesized that the loss of signal corresponded to the half-life degradation of BMP-2. Therefore we consider two types of BMP-2: the BMP-2 which is encapsulated in the gel denoted *g*_*gel*_, and the BMP-2 that is moving in the fluid denoted *g*. We considered that BMP-2 encapsulated can undergo half-life degradation and is also gradually released in the meantime. Both behaviors were modelled by an exponential decay, characterized by $\lambda_{g}^{gel}$ and *λ*_*rel*_ respectively. Thus, the BMP-2 amount in the gel varies according to the following equation:

$$\frac{\partial g_{gel}}{\partial t}=-\lambda_{g}^{gel}g_{gel}-\lambda_{rel}\left(a\right)g_{gel}$$ (7)

where *λ*_*rel*_ (*a*) depends on the normalized amount of the alginate *a* as will be explained later in Eq 13. The amount of BMP-2 released from the hydrogel into the fluid will increase BMP-2 concentration in fluid. The released BMP-2 is now available for cell and in particular can undergo the half-life degradation characterized in Eq (4) with degradation constant $\lambda_{g}^{in\;vivo}$. Thus the complete equilibrium equation with the released and the *in vivo* degradation terms stands as:

$$\frac{\partial g}{\partial t}=\lambda_{rel}g_{gel}-\left(\frac{\partial g_{events}}{\partial t}+\frac{\partial g_{background}}{\partial t}\right)-\frac{\partial g_{degradation}}{\partial t}+\frac{\partial g_{production}}{\partial t}+\frac{\partial g_{diffusion}}{\partial t}$$ (8)

We assumed that a gel exclusively composed of alginate molecules does not permit cell migration. In fact, cells do not naturally adhere to the alginate polymer [84]. However, small RGD peptides can be mixed into the gel to mimic the adhesion sites of the extracellular matrix, thus allowing cell migration inside the gel [84,85]. Although it is possible, we considered the migration rate into the alginate to be low [86,87].

To our knowledge, no alginate-degrading enzyme exists in humans [88]. However, alginate degradation occurs *in vivo* through the loss of ionic cross-links and random hydrolysis of the polymer [85]. As suggested by Boerckel et al. [49], cells can have an indirect influence on alginate hydrogel degradation. As cell and tissue invasion occurs, the ion exchange rate increases, and water penetrates further into the hydrogel, thereby enhancing its degradation.

Based on the previous description, we modelled hydrogel degradation considering that (1) the hydrogel follows an aging degradation behaviour, (2) the cellular influence on degradation is predominant over other effects and (3) the degradation rate is proportional to the number of cells. The model summarized in Eq (9) considers these effects:

$$\frac{\partial a}{\partial t}=\left(\lambda_{deg}^{bulk}-c\;\lambda_{deg}^{cell}\right)\;a\left(t\right)$$ (9)

where *a*(*t*) is the normalized amount of alginate at time $t,\;\frac{\partial a}{\partial t}$ is the degradation rate of alginate at time *t*, *c* is the cell concentration, $\lambda_{deg}^{bulk}$ describes the bulk degradation coefficient and $\lambda_{deg}^{cell}$ describes the degradation promoted by cellular invasion and activity. We assume that two main mechanisms regulate degradation of the alginate. First, bulk degradation which occurs through the hydrolysis of the polymer chains by the water in the gel pores. Second, cell-based degradation [49], the argument being that since alginate chains are degraded by hydrolysis, cellular invasion and activity destabilizes the ionic crosslinking, further exposing the hydrogel chains to hydrolysis. According to the proposed degradation model, the number of cells proportionately increases alginate degradation. However, if the cell population is constant, gel degradation only depends on time [89] and will proceed via the hydrolysis of random polymeric links [85] in an aging process [90].

We also included the regulatory effect of the hydrogel on cell migration and BMP-2 release. Concerning cellular migration, we simulated their motility through Eq (10)

$$\frac{\partial c}{\partial t}=\nabla .\left(D\left(a\right)\nabla c-c\chi \left(a\right)\nabla g\right)$$ (10)

where *c* is the cellular concentration, *g* is the BMP-2 in the fluid, *D*(*a*) is the cell diffusion coefficient and *χ*(*a*) is the chemotactic sensitivity coefficient. The values of *D* and *χ* were defined with a linear dependence on the amount of hydrogel *a*. Therefore

$$D=D_{0}\left(1-a\right)$$ (11)

$$\chi =\chi_{0}\left(1-a\right)$$ (12)

where *D*_0_ and *χ*_0_ corresponds to the diffusion and chemotactic coefficients when the gel matrix does not exist. We considered here that migration is heavily impaired by alginate, although a weak random migration is allowed. In fact, cells are not keen on alginate and when presented with the alginate gel alone the migration is very weak, as Boerckel et al. showed [49]. It can also be seen that dense gels can impede cells from migrating even in the presence of chemical queues. Nonetheless, this obstacle is gradually reduced as the hydrogel degradates.

Also concerning cell migration, MSCs were not allowed to migrate through mineralized matrix [31]. It may happen however, that during the defect infilling, both non-mineralized and mineralized tissue temporarily inhabit the same space for a limited time period. Therefore, we assumed that MSCs are not allowed to migrate through a certain volume if mineralized tissue is the main tissue in that volume.

Finally, we also assumed that the way BMP-2 is released would have to be influenced by the amount of alginate. The reasoning behind this assumption comes from the idea that as the alginate is degraded, its contact surface increases thus increasing the release rate of BMP-2. Accordingly, the degradation constant *λ*_*rel*_ is defined with a linear dependency on the amount of alginate hydrogel:

$$\lambda_{rel}=\lambda_{rel}^{0}\left(1-a\right)$$ (13)

where $\lambda_{rel}^{0}$ is a constant that quantifies the release rate of BMP-2.

### 2.4. Use of BMP-2 to heal critical size fractures

We aim to study, the healing effect of BMP-2 on critical fractures *in silico*. We have shown the influence of BMP-2 on cell behaviour in terms of fold increases relative to cells not treated with BMP-2. Assuming that previous mechanoregulatory models [31,57] implicitly consider BMP-2 only at the physiological level, we can update these models by fold increasing the mechanical effects on cells and tissues, given BMP-2 influence.

The main idea behind the coupling is stated by several authors who stress that BMP-2 is a modulating factor [47,52,63,68]. In fact, BMP-2 can play a major role in modulating the sequential events leading to bone formation [58]. Based on this *motto*, the fold increase in proliferation, migration, differentiation, maturation and matrix production were determined by the modulating functions presented in Figs 1–4, respectively.

The suggested BMP-2 modulation can be applied to any mechanoregulatory model, as long as it considers the behaviour to be modulated. In this work, we used the mechanistic model presented by Gómez-Benito et al. [31] and García-Aznar et al. [57], as it already considers all modulated effects (i.e., proliferation, migration, differentiation, maturation and matrix production.

**Fig 5:**
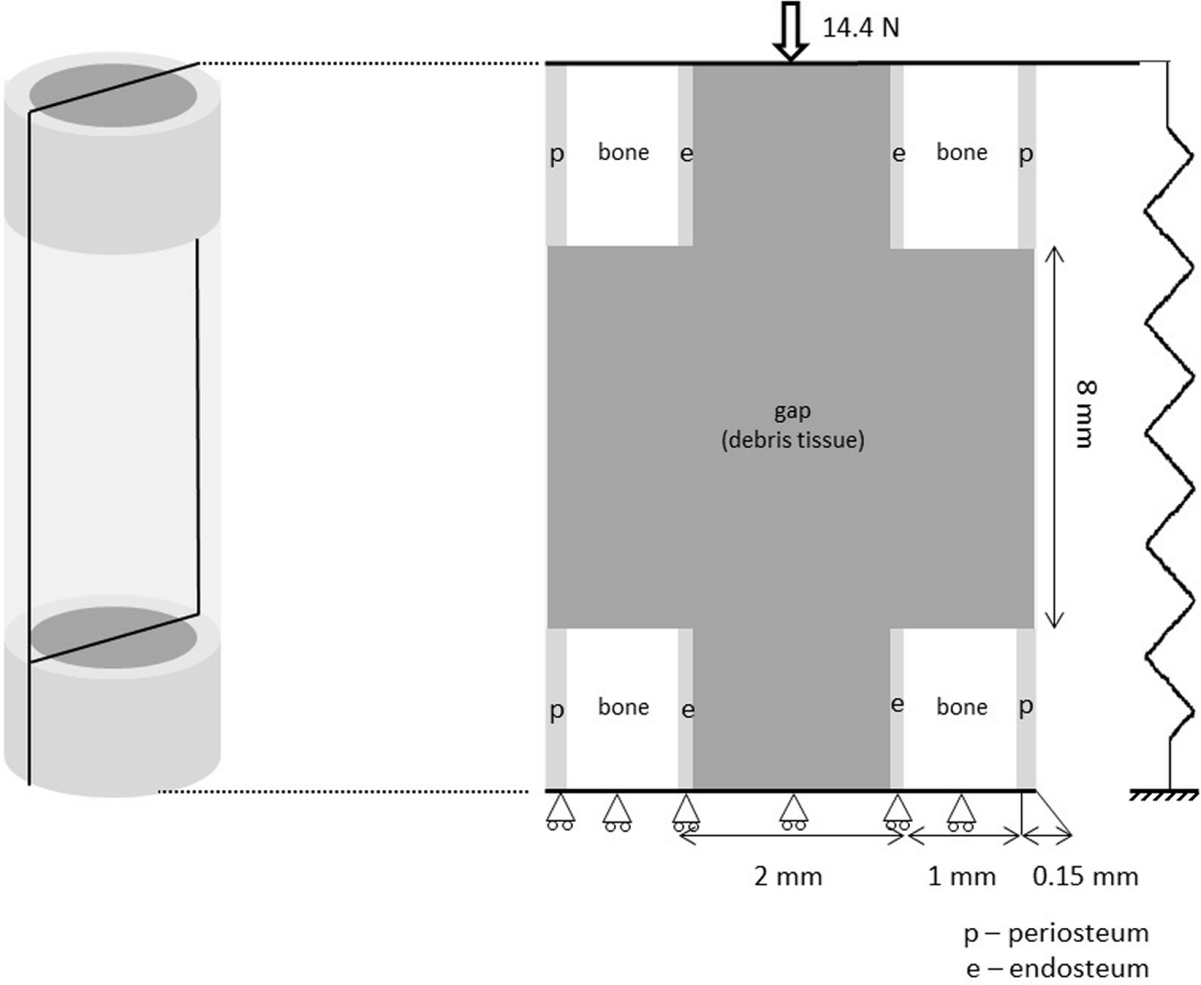
Rat idealised geometry with the respective dimensions, different bone parts and load.

To evaluate the predictive ability of the model, we simulate the *in vivo* experiments of Boerckel et al. [49], who investigated the effect of BMP-2, released by an alginate hydrogel, in an 8 *mm* critical bone defect, in the diaphysis of a rat femur. The geometry of the rat femur diaphysis is considered as an idealized cylinder, to which only axial loads are applied. Given the cylindrical symmetry, our study was performed on one longitudinal section. From a mechanical perspective we assumed 2D-plane strain [91, 92]. From a mass diffusion perspective, the 2D model considered a unitary thickness, meaning that every point within the model domain is surrounded by a control volume. Thus we kept using volume units (*ng*/*cm* ^3^) while working with BMP-2 concentrations. The femur geometry is presented in Fig 5 and includes the cortical bone, endosteum, periosteum and gap, which was set at 8 *mm* [49].

We applied an axial load of 14.4 *N* which corresponds to the maximum axial load during the rat gait [93]. We used a fixator with a stiffness of 277 *N*. *mm* ^−1^ estimated from the polysulfone fixator used *in vivo* by Boerckel et al. [49]. In agreement with the original model by Gómez-Benito et al. [31], as bone healing occurs, cells proliferate, migrate, differentiate and produce biological tissues. The tissues considered were debris tissue, granulation tissue, cartilage, fibrous tissue, woven bone and cortical bone. All tissues were assumed to be isotropic poroelastic biphasic materials. Their properties are summarised in Table 1.

**Table 1:** Poroelastic mechanical properties considered for the different tissues [31,94–96] and for the alginate hydrogel.

*Tissue*	*E* (*MPa*)	*ν*	*k* (*mm* ^2^)
Debris tissue	1.85	0.048	7 × 10^−12^
Granulation tissue	7.79	0.048	1 × 10^−14^
Cartilage tissue	27.05	0.103	5 × 10^−15^
Calcified cartilage	57.05	0.108	5 × 10^−15^
Fibrous tissue	80.07	0.127	1 × 10^−14^
Woven bone	982.48	0.295	1 × 10^−17^
Cortical bone	20 × 10^3^	0.3	1 × 10^−17^
Alginate hydrogel	0.05 [98,101]	0.048	7 × 10^−12^

We assumed that alginate hydrogel is a poroelastic biphasic material. The Poisson’s ratio and the permeability were considered to be within the same order of magnitude of those of debris tissue [97], and the Young modulus was set at *E*_*Hg*_ = 0.05 *MPa* [98–101].

Since the mechano-chemical model proposed is an extension of the mechanical model proposed by Gómez-Benito et al. [31], the mechanical stimulus is computed as described in the work of Gómez-Benito et al. [31]. Briefly, at each iteration a finite element mechanical analysis is used to compute the mechanical stimulus. Tissues are considered poroelastic, hence described by an elastic modulus, a Poisson’s coefficient and a permeability coefficient. Knowing the tissue distribution and their material properties, the material constants at each Gauss point are determined with an average weighted by the tissue fraction, as it was proposed by Gómez-Bénito et al. [31]. Once the material properties are defined we applied the external loads. From the mechanical analysis the principal strains at each point are extracted and used to compute the mechanical stimulus (second invariant of the deviatoric strain tensor). Given the computation of the mechanical stimulus, we now have the necessary information to run the mechanistic model.

We quantified the effects of BMP-2 on cell behavior in order to evaluate the use of BMP-2 in large bone defects. With this purpose, we defined a series of *in silico* experiments. For validation purposes and following the *in vivo* bone healing experiment by O’Neil et al. [102], we tested the proposed mechano-chemical model in a regular 2*mm* gap fracture. The aim of this study was to verify that BMP-2 at normal physiological concentrations, without any exogenous source, did not alter regular the regular simulation of bone healing. We next tested different conditions for large bone defects. The first condition corresponded to a control experiment, in which the gap was empty and contained no hydrogel or BMP-2 supply. In the second experiment, following the *in vivo* approach by Boerckel et al. [49], the gap was filled with alginate hydrogel alone, and no BMP-2 was provided. In the third and final experiment, following again the *in vivo* approach by Boerckel et al. [49], we implanted the alginate hydrogel soaked with 5 *μg* of BMP-2 into the bone defect. Besides the 5*μg* dose, and from a quantitative perspective only, other BMP-2 doses were also tested (0.1*μg*, 0.5*μg*, 1*μg* and 2.5*μg*), in order to further evaluate the quantitative predictive potential of the model.

## Results

### 3.1. First experiment: validation for a 2*mm* gap

**Fig 6:**
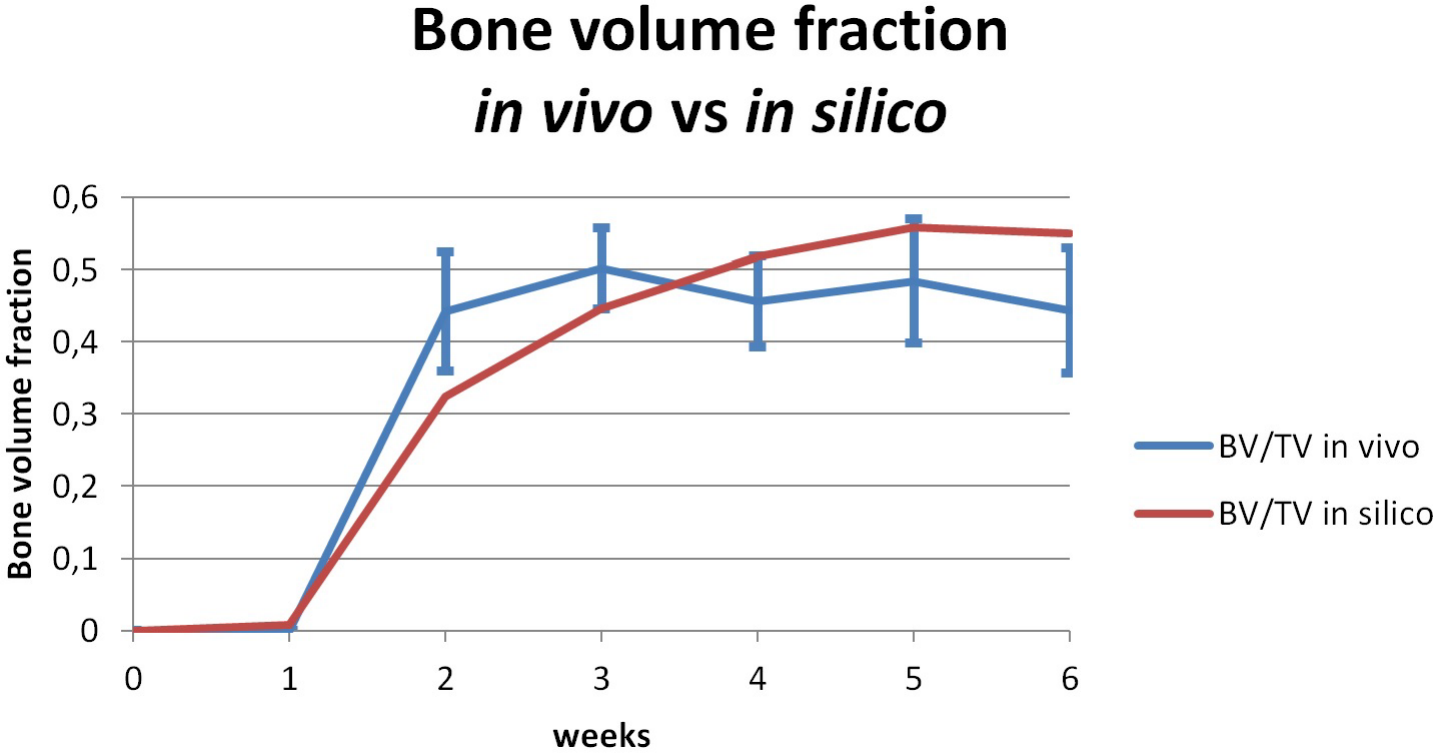
*In silico* results for a small gap (*2mm*). The evolution of callus shape and bone tissue filling the callus and gap regions

For the 2*mm* gap fracture, and following the *in vivo* bone healing experiment by O’Neil et al. [102], we studied the evolution of bone formation inside the callus and gap regions (Fig 6).

From a qualitative perspective, an exhaustive description of healthy bone healing as well as of all the features captured by the 2*mm* gap simulation is provided in the S1 File. Briefly, and concerning bone tissue formation, we observed that the callus progressively appeared during the first two weeks, intramembranous ossification occurred in the periosteum and new bone tissue slowly appeared far from the fracture site. From week 3 until week 5, the callus was progressively invaded by bone: endochondral ossification takes place and the hard callus was formed. By the end of week 5, the callus was fully filled with new bone tissue and began to act as a buttress, improving gap stability. By week 6, the increased stability brought by the hard callus allowed bone tissue to fill the gap, and the bone continuity was restored.

**Fig 7:**
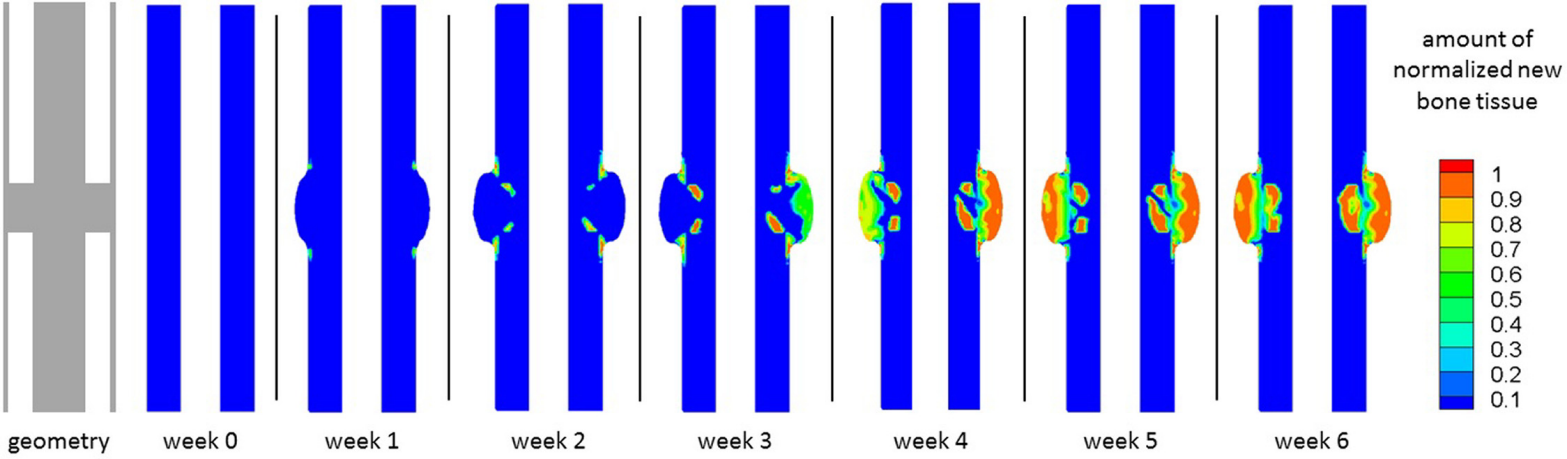
Evolution of normalized new bone tissue *in vivo* and *in silico*. fraction measure (blue) *in vivo* [**102**] and predicted in silico (red) for a rat fracture model of 2mm

From a quantitative perspective, Fig 7 shows the predicted amount of normalized new bone formed during the 2*mm* fracture healing. There, the *in silico* results are plotted against the normalized amount of bone measured by O’Neil et al. [102] (also during a 2*mm* fracture healing). As we can observe, the amount of predicted bone closely follows the amount of measured bone *in vivo*.

Together, Figs 6 and 7 show that the model predictions mirror the description of healthy bone healing observed *in vivo* from both a qualitative and a quantitative perspective [102,103].

### 3.2. Second experiment: predictions of BMP-2 based healing of a large bone defect

**Fig 8:**
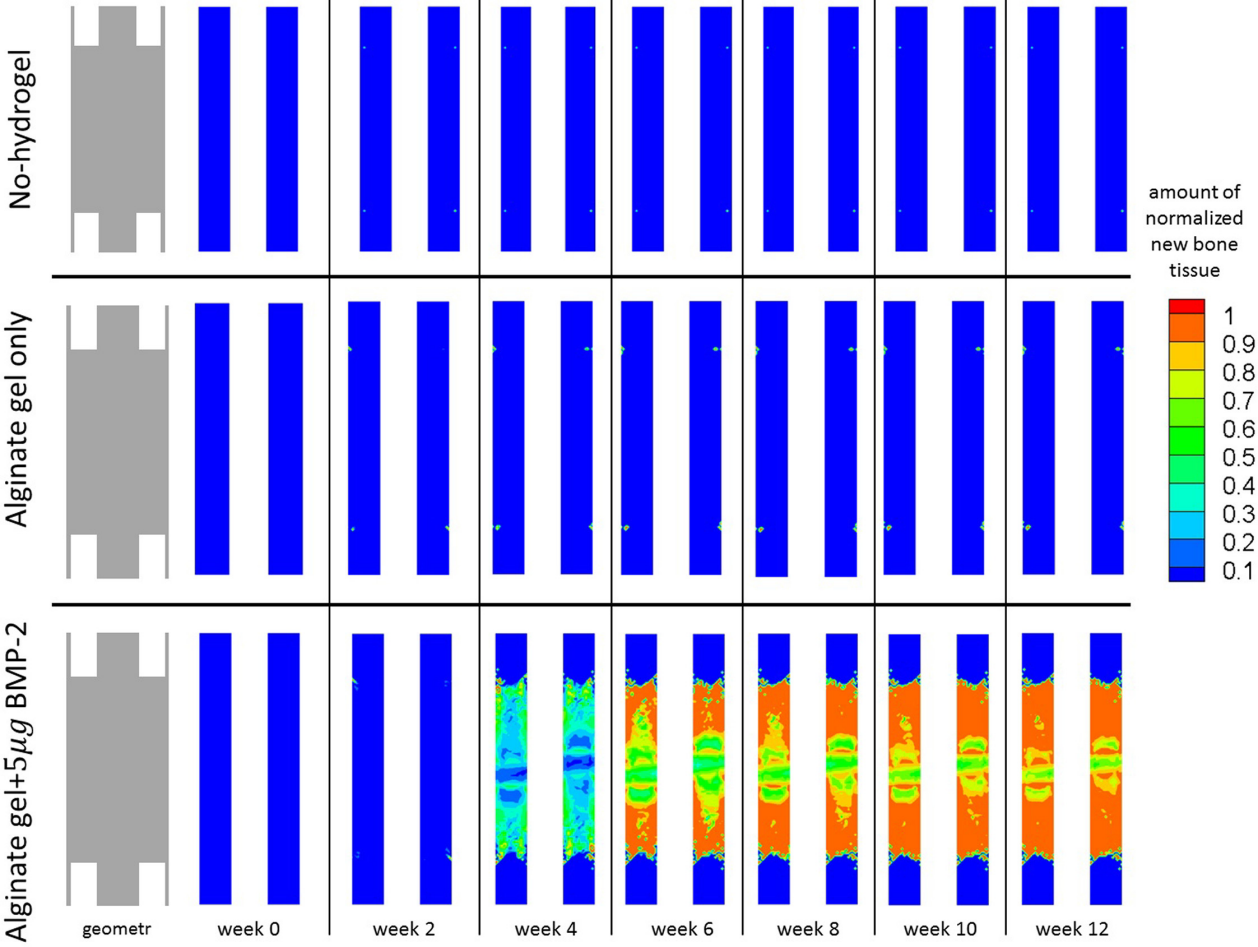
Evolution of new bone tissue. Evolution of the normalized amount of new bone tissue inside the bone defect over 12 weeks, when: no hydrogel was placed inside the bone defect (top); an alginate hydrogel was placed inside the bone defect (middle), and an alginate hydrogel with *5μg* of BMP-2 (bottom) was placed inside the bone defect.

**Fig 9:**
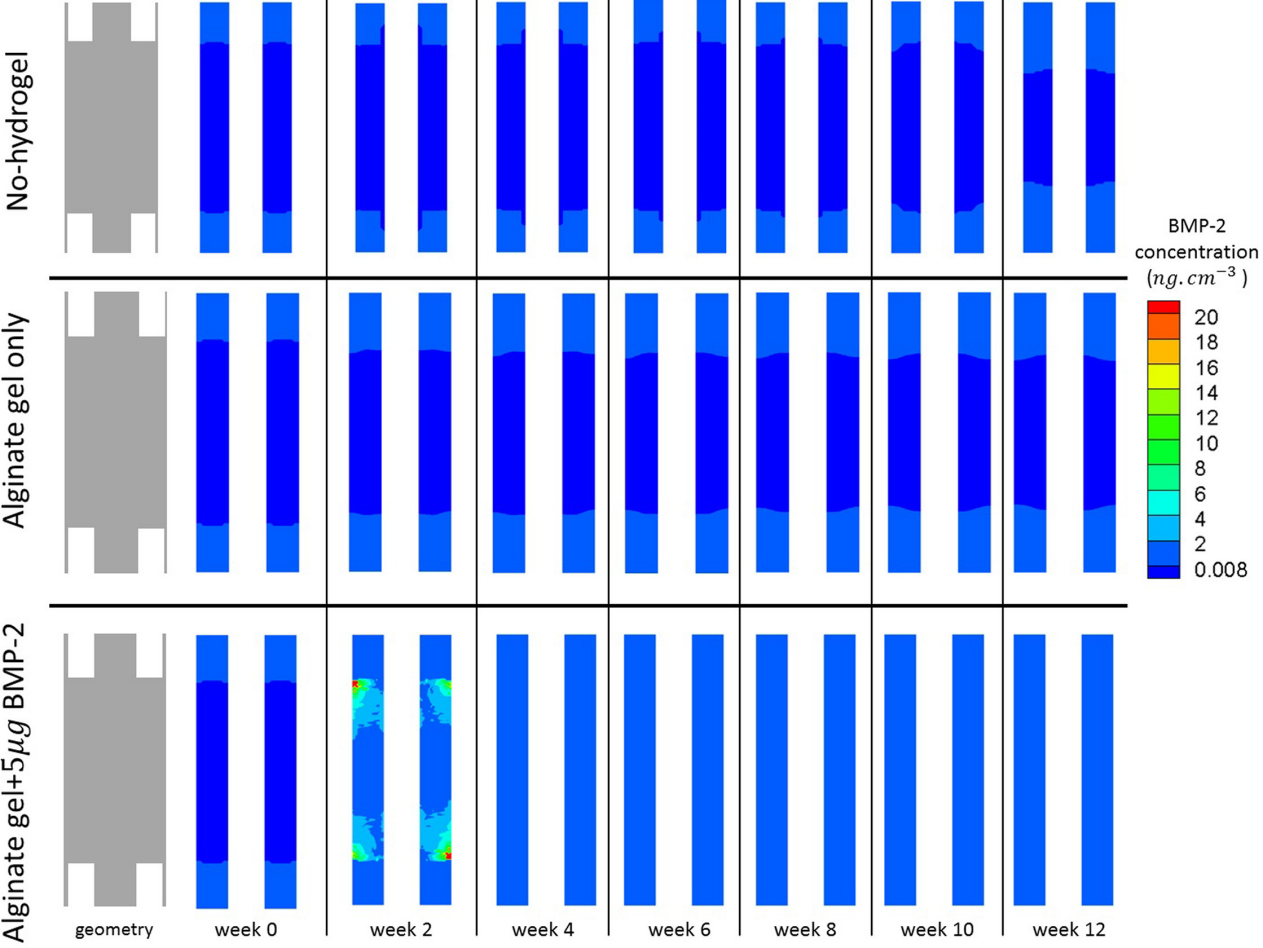
Evolution of BMP-2 concentration. Evolution of BMP-2 concentration inside the bone defect over 12 weeks, when: no hydrogel was placed inside the bone defect (top); an alginate hydrogel was placed inside the bone defect (middle), and an alginate hydrogel with *5μg* of BMP-2 (bottom) was placed inside the bone defect.

For each of the remaining three experiments, we studied the evolution of bone formation inside the gap as well as the evolution of 5*μg* dose BMP-2 release over a period of 12 weeks [49]. These results are presented in Figs 8 and 9 respectively.

With respect to new bone formation (Fig 8), bone appeared and filled the gap only when both the hydrogel and BMP-2 were used. For the cases receiving hydrogel alone or no hydrogel, no new bone formation was detected.

When both hydrogel and BMP-2 were used (5*μg* dose), bone tissue formation inside the defect was low during the first month. It is just at the fourth week that the produced amount of new bone tissue crosses the 10% threshold. Thereafter, the amount of bone gradually increased inside the gap. By the end of week 12, new bone tissue occupied approximately 85% of the defect and the bone tissue distribution tended to be greater (around 90%) in the regions next to cortical bone.

The BMP-2 concentration was either low and close to the physiological levels or almost non-existent for both the no-hydrogel and alginate only cases (Fig 9). However, when hydrogel and BMP-2 (5*μg* dose) were used simultaneously, we observed an initial burst of BMP-2, which lasted for the first two weeks and then gradually decreases until week 6. From week 6 until the end of week 12, the BMP-2 concentration remained within the physiological range.

The evolution of alginate was also analyzed at week 0 and week 12 (Fig 9). At week 0, the gap was completely filled with the hydrogel. By week 12, the hydrogel remained nearly intact when alginate alone was used, but the amount of alginate was severely reduced when BMP-2 was present. In the latter case, approximately 10% of the hydrogel remained inside the gap. Although some small isles with a slightly greater amount of alginate persisted, the hydrogel was more degraded in the regions adjacent to the cortical bone than in the peripheral regions of the defect.

**Fig 10:**
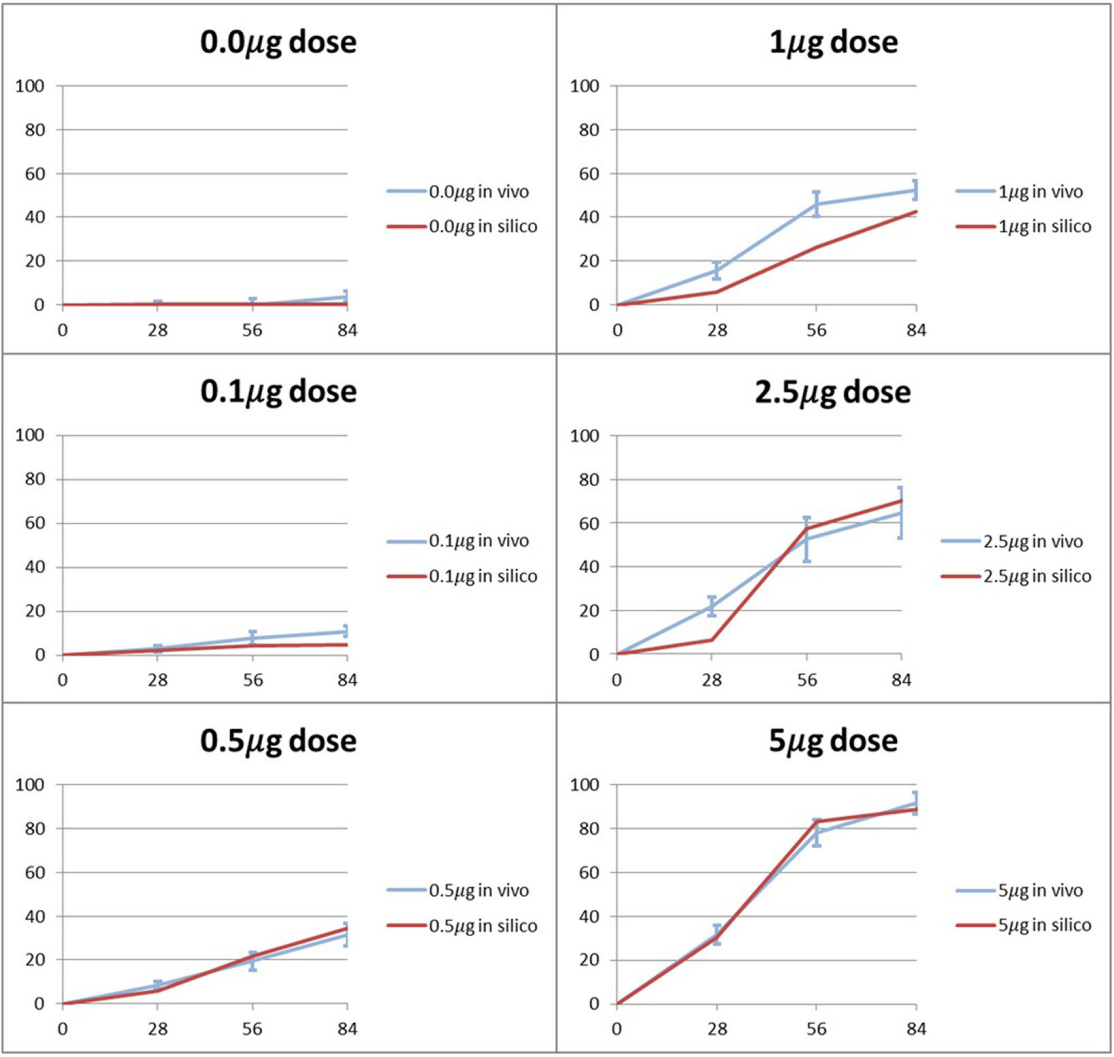
Evolution of bone defect filling with new bone tissue *in vivo* and *in silico*. Percentage of the defect volume filled with bone tissue, for different doses of BMP-2.

From a quantitative perspective, in Fig 10 we show the amount of bone predicted *in silico*, for the different BMP-2 doses (0.0*μg*, 0.1*μg*, 0.5*μg*, 1*μg*, 2.5*μg*, 5*μg*), against the bone amount measured *in vivo* by Boerckel et al. [49] for the same BMP-2 doses. One can observe that for all cases, the curves predicting bone formation *in silico* follow the bone amount measured *in vivo*.

Globally, the *in silico* curves presented in Fig 10 follows the tendency obtained by the *in vivo* curves. Briefly, during the first 28 days, there is an initial increase in the bone amount. Between 28 and 56 days, the bone production rate increases. Finally, from day 56 to day 84, the bone production rate decelerates until the end of the simulation.

Also, we can observe in Fig 10 that the *in silico* simulations capture the progressive increase in bone volume with BMP-2 dose: the release of a greater BMP-2 dose is accompanied by the formation of a greater amount of bone in the defect. In fact, according to the model proposed in this work, bone tissue production (and mineralization) depends linearly: on the number of bone cells (as proposed by Gómez-Benito et al. [31]) and on the chemical stimulus provided by BMP-2 (Fig 4). On the one hand, a greater BMP-2 dose attracts more cells through chemotaxis. On the other hand, a greater BMP-2 dose also improves the bone tissue production rate. Together, these two effects explain why a greater BMP-2 dose promotes defect infill with bone tissue. To see the bone tissue distributions inside the defect, please consult the S2 File.

At the end of the simulations, we found that the more accurate prediction was made for the 0.1*μg* case whose predicted amount of bone deviates from the *in vivo* measurements by 0.4%. The worst prediction corresponds to the 1.0*μg* case which deviates from the *in vivo* experiments by 10%. Nevertheless, the latter margin can be considered acceptable from an engineering and biological point of view. In addition, the error found corresponds to an under prediction of bone volume, in circumstances (1.0*μg* dose) where defect non-union still is observed *in vivo*.

However, when high doses are used (2.5*μg* and 5*μg*) bone bridging occurs and the amount of bone predicted is more accurate (5.6% and 2.9% respectively). Actually, it seems that for intermediary doses a lower production of bone volume can occur, despite the fact that defect non-union keeps being predicted. Therefore, the model seems to provide a conservative prediction: it clearly shows that a larger dose is effectively required for defect bridging.

## Discussion

After simulating the healthy bone healing in a 2*mm* fracture, we simulated the experiments for large bone defects. To verify the quality of the defect filling, Boerckel et al. [49] assessed some qualitative indicators such as defect bridging, bone volume, torsion to failure and made histological observations of cell and tissue invasion into the defect. *In vivo*, Boerckel et al. [49] did not observe defect bridging when a hydrogel alone was placed into the defect. Nevertheless, when gel containing 5*μg* of BMP-2 was implanted, the bone defect was effectively bridged with bone tissue. Our *in silico* results are very similar (Fig 8). Bone bridging occurred when hydrogel and BMP-2 were added and no bridging occurred when hydrogel alone was implanted. Notably, bone tends to be more densely distributed next to the cortical region. Because our model assumes that the periosteum is the main progenitor cell source, increased cell accumulation also increases bone tissue production in this region of the defect.

When neither hydrogel nor BMP-2 was added into the defect, no bridging was observed *in silico*. This result was somewhat expected: in the absence of a real support that allows for cell adhesion, cells cannot migrate. It should be noted however that the no-hydrogel case presented in Fig 8, was not tried by Boerckel et al. [49]. Instead it is used here as a control experiment of the model presented by Gómez-Benito et al. [31] in order to verify whether or not large bone defects are within the range of validity of the referred model. Without any cellular support or molecular cue, we would expect cellular invasion and ECM production into the gap to be almost non-existent. Indeed, we did not observe any cell invasion or ECM formation; in particular, bone tissue (Fig 8) did not appear over the twelve week period. Combining the observations of the 2*mm* gap experiment and the no-hydrogel case for an 8*mm* defect, we noticed that for both regular size gaps (2*mm*) and large bone defects (8*mm*) the proposed mechano-chemical model correctly predicts bone union and non-union, respectively. Therefore, the model proposed here is able to predict different healing patterns for different gap dimensions.

Comparing bone tissue formation between the 2*mm* fracture and the 8*mm* defect with BMP-2 soaked hydrogel, we notice that bone formation in the former case follows the formation of the external periosteal callus. The mechanistic model proposed by Gómez-Benito et al. [31], which is at the basis of our mechano-chemical model, considers that this domain variation is caused by two main phenomena: increased cell proliferation (in particular MSCs) and cartilage hypertrophy. In our numerical simulations, domain variation is not observed for the large defect. On the one hand the stability provided by the external fixator is high, thus enhancing a quick differentiation of MSCs into bone cells which do not have enough time to proliferate. On the other hand, whenever cartilage appears, it is quickly replaced by bone. Hence, there is never a significant amount of cartilage nor enough time for hypertrophy to change the model geometry.

Bone volume and torsion to failure, are quantitative indicators of defect bridging. Bone volume can be calculated, as shown in Fig 9. Although bone volume is formed more rapidly *in vivo* than *in silico*, bone volume inside the defect *in vivo* and *in silico* is very similar after twelve weeks. *In silico*, the global evolution of bone tissue formation inside the gap is quantitatively estimated. The sometimes slower initial bone formation may be due to a simplification that ignores osteoprogenitors cells others than MSCs [31,57]. In fact, if sources such as bone marrow and muscle osteoprogenitors were considered [104], more bone cells would be present inside the defect, which would accelerate initial bone tissue production inside the gap.

**Fig 11:**
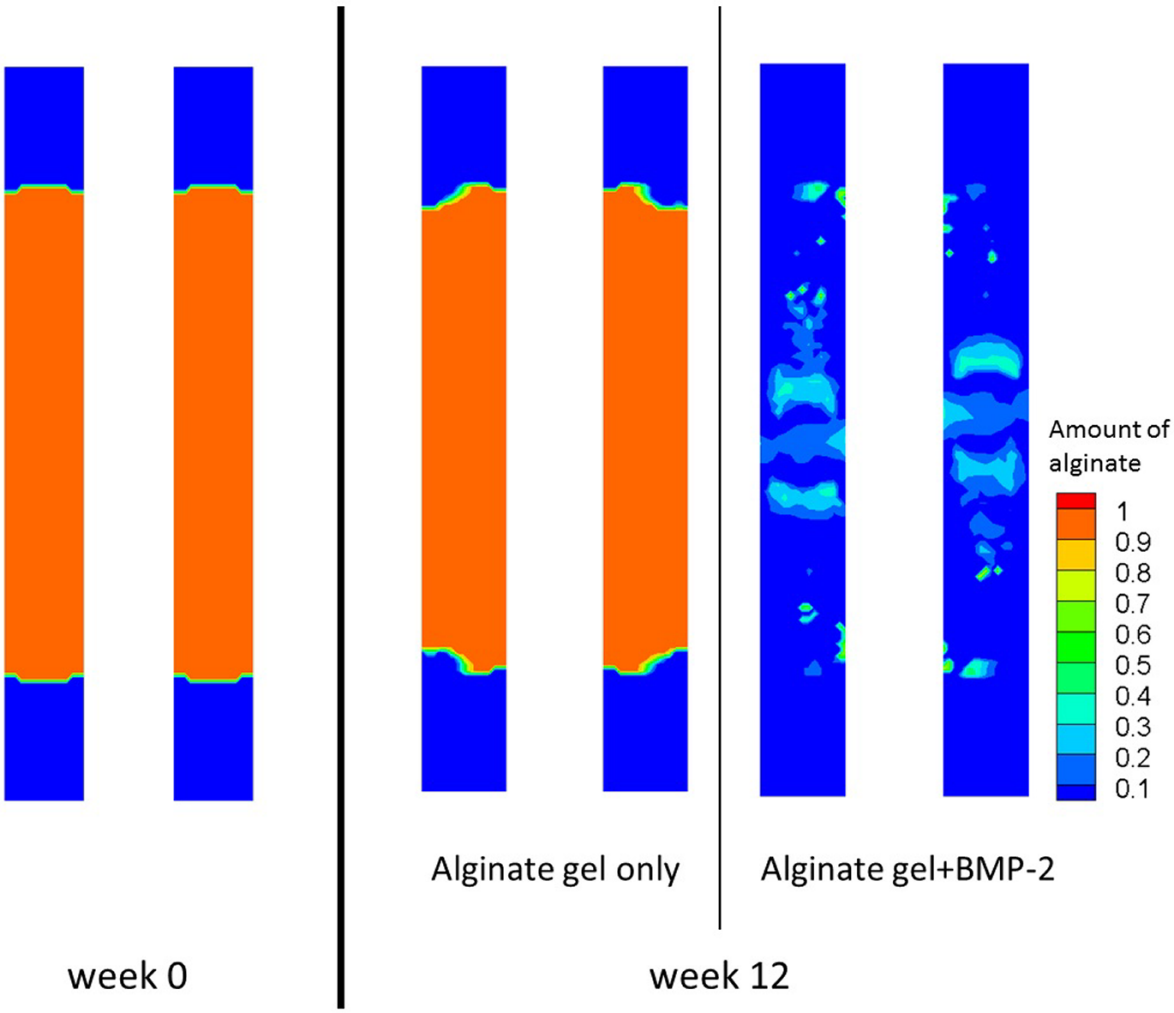
Alginate hydrogel normalised distribution inside the bone defect. Alginate hydrogel normalised distribution inside the bone defect after 12 weeks, for the case where alginate alone is implanted inside the bone defect and for the case where alginate with *5μg* of BMP-2 is implanted inside the bone defect.

Boerckel et al. [49] found that even when bridging occurs, there is cellular and tissue invasion and a significant, yet incomplete, degradation of the hydrogel. Similarly, we observed *in silico* cell invasion whenever the hydrogel was implanted. Through hydrogel degradation (Fig 11), we observed the indirect effect of invading cells. When BMP-2 was used, hydrogel degradation was increased, reflecting increased cellular invasion. As observed *in vivo*, the alginate never fully degraded and the hydrogel remaining *in silico* was analogous to the hydrogels islets observed *in vivo*.

So far we proposed a mechano-chemical model that incorporates the effect of BMP-2 on cell events which regulate tissue regeneration. While Moore et al. [105] focused on the stimulus of endogenous BMP-2, which depended on the mechanical strain, in this work the BMP-2 stimulus provided by an exogenous source was considered as well as its modulatory interplay with the mechanical stimulus. To the best of our knowledge, this is the first *in silico* model that considers the bone induction properties of exogenous BMP-2 to correctly predict bone defect bridging. The developed model also predicts the amount of bone and its distribution in the defect at twelve weeks.

Nevertheless, we have to keep in mind that our model presents some limitations, which have been carefully analyzed. First, we observed that defect bridging occurred more slowly in the first few weeks (Fig 11), which could be attributed to other osteoprogenitors cell sources that were not considered, such as bone marrow and the surrounding soft tissues. Secondly, by not considering the surrounding soft tissues and the MSCs provided by them, certain phenomena observed *in vivo* such as ectopic bone formation cannot be recovered by the *in silico* simulations of this model.

Thirdly, the 2D geometry used in this work also presents some limitations. For instance, in their *in vivo* study, Boerckel et al. [49] measured the stiffness to failure. The current model was defined using 2D-plane strain to represent the symmetry plane. However, to measure this last mechanical value, the current 2D model would have to be updated to a 3D model to provide greater insight and further validation. Furthermore, we underestimate the healing of the medullary canal. This simplification is based on the assumption that two main MSCs sources contribute to bone formation in the cortical gap defect: the periosteum and the endosteum sources. Our assumption is that cells coming from the periosteum domain are the ones that are going to contribute the most to the cortical gap regeneration, while we do not consider the contribution of endosteal cells to the regeneration of the cortex. Therefore we consider that cells from the endosteum mainly affect bone formation in the endosteum region. Certainly, the underestimation of the endosteal region can slightly alter the mechanical environment; however, we have developed different simulations verifying that the mechanical contribution of the endosteal callus is not significant. Therefore, the rationale for not including the intramedullary space is not only mechanical [106] but more based on how cells distribute around the defect.

In our model, we did not consider bone remodeling phenomena. We took this simplification for two reasons: first, bone remodeling can take up to several years [107] and the time period considered reaches only 12 weeks, a time frame when remodeling does not play a major role yet. Second, as suggested by Boerckel et al. [49], the presence of the alginate impairs an already slow bone remodeling process. Therefore, this assumption is adequate given that we focus on the short-term response. Nevertheless, future updates of the presented model could incorporate this effect in order to predict both the bone volume inside the defect and its mechanical properties.

In contrast to previous qualitative healing models that incorporate growth factors, we have taken a quantitative approach instead of a qualitative one [25,31,32,34,57]. Although this new perspective is based on experimental data and thus requires fewer parameters, it always depends on the data available in the literature. For instance, the influence of high concentrations of BMP-2 was only available for cell proliferative behaviour (Fig 1), so we assumed an upper asymptote for chondrocyte hypertrophy at high concentrations. Following the same rationale, we only considered the effects of BMP-2 on cell behaviours that were described in the literature. A description of the effects of BMP-2 on fibroblasts, for example, was not found in the literature and, therefore, was not considered. In this context a sensitive analysis for the modulatory curves was done (see S3 File) and the results suggest that alternative curves did not cause a significant behavioral change. Nevertheless, whenever new data are available, it can easily be incorporated into the model to update the proposed modulation curves (Figs 1–4) or even to suggest new ones for new cellular behaviors.

Moreover, we must also stress that BMP-2 is just one in the myriad of growth factors involved in bone healing. We focused on BMP-2 specifically because it is considered by many authors to be one of the most important growth factors for bone healing [45,47] and bone tissue regeneration [14,15]. Given the availability of quantitative data, other growth factors can also be considered in the future to improve our approach.

In this work, our initial working hypothesis is that the mechanical stimulus interacting with the biochemical stimulus can be a useful approach to study this complex system where both stimuli play an important regulatory role. Thus, BMP-2 was introduced following what several authors in the literature consider to be its modulatory effect [52,63,68,70]. From a mathematical point of view, this assumption is translated as the product between mechanical and chemical stimuli dependent terms.

## Conclusion

Previous *in silico* healing studies have shown that mechano-regulatory models are useful for qualitatively predicting the evolution of bone healing in small gap fractures [29,31,32,34,57]. Following the same mechanical perspective, recent studies on large bone defects have focused on the mechanical performance and stability due to rigid scaffold constructs [6,38,39,41]. In this work, we proposed a novel *in silico* mechano-chemical approach that puts a greater emphasis on a growth factor, specifically BMP-2, as a chemical stimulus. This was achieved by collecting quantitative experimental data concerning the effect of the growth factor on cells and tissues. This model brings up the opportunity of testing *in silico* the recent advances made on large bone defects healing, which show an enhanced defect bridging through the use of BMP-2 [108]. Importantly, we successfully predicted the healing and non-healing of a large bone defect, with BMP-2 delivery rather than a rigid scaffold. Moreover, our results showed a good qualitative and quantitative agreement with previous *in vivo* studies [49]. This novel *in silico* tool can provide further insight for bone tissue regeneration strategies to better determine the necessary doses of BMP-2 that guarantees bone healing, to optimize the release kinetics so that bridging is achieved with as less BMP-2 as possible thus enhancing bone healing [108].

## Appendix 1

In this appendix we define the constants and parameters used in this paper.

- *g*_*min*_ = 0.008 *ng*. *cm* ^−3^ is the low boundary of the *in vivo* concentration range of BMP-2
- *D*_*g*_ = 0.2 *mm* ^2^ *day* ^−1^ is the diffusion coefficient of BMP-2 in the alginate hydrogel [108], *D*_*g*_ = 8.64 *mm* ^2^ *day* ^−1^ is the diffusion coefficient of BMP-2 *in vivo* [109] and *α*_*prod*_ = 2 × 10^−9^ *ng*. *ml* ^−1^ *cell* ^−1^ *day* ^−1^, *γ =* 15 (*ng*/*mL*)^−1^ and *γ*_0_ = 0.01 are three parameters that define the BMP-2 production function
- *V*_*max*_ = (*c*_*s*_ + *c*_*b*_) × 1.43 × 10^−7^ *ng*. *cm* ^−3^.*day* ^−1^.*cell* ^−1^ is the maximum rate at which BMP-2 is consumed by cells [73]. MSC and bone cells are assumed to be the main contributors to BMP-2 consumption [20,45,47,68,74].
- $K_{m}^{a}=11.01\;ng.cm^{-3}$ is a constant analogous to the Michaelis constant [73]
- *λ*_*r*_ = 0.217 *day* ^−1^ is the decay constant in the BMP-2 release profile [49]
- $\lambda_{deg}^{bulk}=5\times 10^{-4}day^{-1}$ is the hydrogel degradation constant for bulk degradation and $\lambda_{deg}^{cell}=6.5\times 10^{-6}day^{-1}.cell^{-1}$ is the degradation constant due to cell influence
- $\lambda_{rel}^{0}=0.027\;day^{-1}$ characterizes de the release rate of BMP-2 from the hydrogel
- $t_{1/2}^{gel}=3.25\;days$ is the half-life time of BMP-2 encapsulated inside the hydrogel and $t_{1/2}^{in\;vivo}=0.42\;days$ is the half-life time of BMP-2 *in vivo* [75].

## Supporting Information

S1 File Simulation of the normal bone healing in a murine 2mm fracture. (PDF)

S2 File Bone distribution for several BMP-2 doses. (PDF)

S3 File Sensitivity analysis of the modulatory functions. (PDF)
