# Supplementary material for: In silico Mechano-Chemical Model of Bone Healing for the Regeneration of Critical Defects: The Effect of BMP-2
Source: PLoS One. 2015 Jun 4;10(6):e0127722. doi: 10.1371/journal.pone.0127722 (PMC4456173; doi:10.1371/journal.pone.0127722)
Supplement: S1 File — (PDF) [file pone.0127722.s001.pdf]

Support Information 1

As underlined in the Discussion section, we reproduced *in silico* the bone healing results found by O'Neil et al. [97] for a $2mm$ defect, in order to ensure that the incorporation of BMP-2 into Gómez-Benito et al. [31] model did not change regular bone healing.

The results presented in Fig. 6 are expanded here in order to provide further validation and a full description of the healing mechanisms. The fully detailed results are shown in Fig. S1 with the evolution of granulation tissue, cartilage, fibrous tissue and new bone in the callus and the fracture. Note that the original mechanical model of bone healing, by Gómez-Benito et al. [31], predicts similar results, meaning that BMP-2 at normal physiological concentrations does not change the Gómez-Benito et al. bone healing predictions [31].

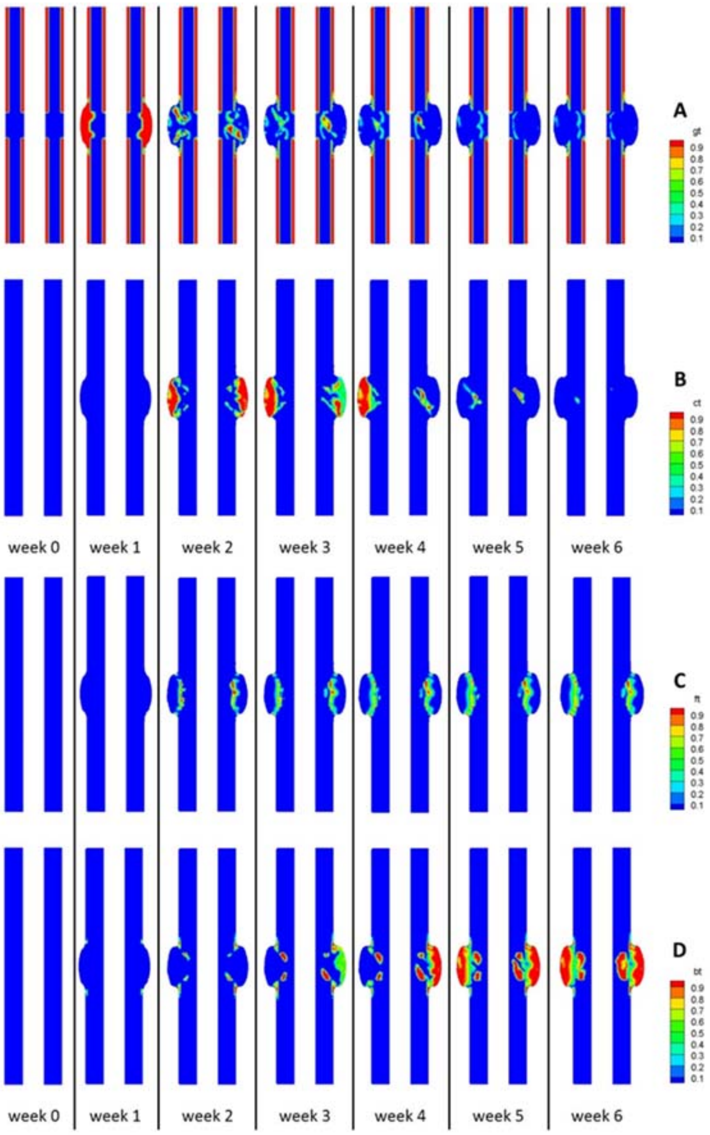

Fig. S1- **Normal bone healing in a murine 2mm fracture**. Tissue distribution and evolution along the healing time course. (A) evolution and distribution of normalized amount of granulation tissue, (B) evolution and distribution of cartilage tissue, (C) evolution and distribution of fibrous tissue, (D) evolution and distribution of new bone tissue.

As observed in Fig. S1, before bone healing starts (week 0), none of the above mentioned tissues are present in the fracture. As a consequence of bone trauma, the main material existing at that time point in the gap is debris tissue. During the first healing stage, an inflammatory reaction is triggered whose role is the cleaning of all the debris. The recruitment of cells, such as macrophages, supports this process which is closely followed by granulation tissue production by mesenchymal stem cells (MSC). MSCs have the periosteum as their main source although other surrounding soft tissues also provide the system with MSCs. It is in this first stage, that granulation tissue appears in mice (Fig. S1, week 1).

Following the increment in stability provided by the newly formed granulation tissue, MSCs differentiate into chondrocytes which are responsible for cartilage production. This is the second stage of the healing process and it is characterized by the buildup of a cartilage callus, also commonly known as soft callus (Fig. S1, week 2). The less stable regions however tend to have an increased concentration of fibrous tissue instead of cartilage tissue.

Simultaneously, intramembranous ossification takes place at the callus periosteal extremes (Fig. S1, week 2 and 3). This works as a foothold for the endochondral ossification that later on fill with woven bone (Fig. S1 week 3, 4 and 5). This later formation of new bone tissue constitutes the third stage of healing, in which the hard callus forms this bony buttress that further enhances the mechanical stability of the healing bone.

The fourth and last healing stage typically described comprises bone modelling and remodeling within the callus. There, bone modelling turns woven bone into cortical bone while the callus is gradually resorbed. This last stage is well observed in the original work developed by O’Neil et al. [97], mainly after week 6. The model proposed by Gómez-Benito et al. [31] only consider the first three healing stages as described before, and thus the bone healing simulated in Fig. S1 only goes till week 6.
