# Supplementary material for: In silico Mechano-Chemical Model of Bone Healing for the Regeneration of Critical Defects: The Effect of BMP-2
Source: PLoS One. 2015 Jun 4;10(6):e0127722. doi: 10.1371/journal.pone.0127722 (PMC4456173; doi:10.1371/journal.pone.0127722)
Supplement: S2 File — (PDF) [file pone.0127722.s002.pdf]

Support Information 2

In Fig. S2, we can observe the evolution of bone tissue inside the defect during the simulation.

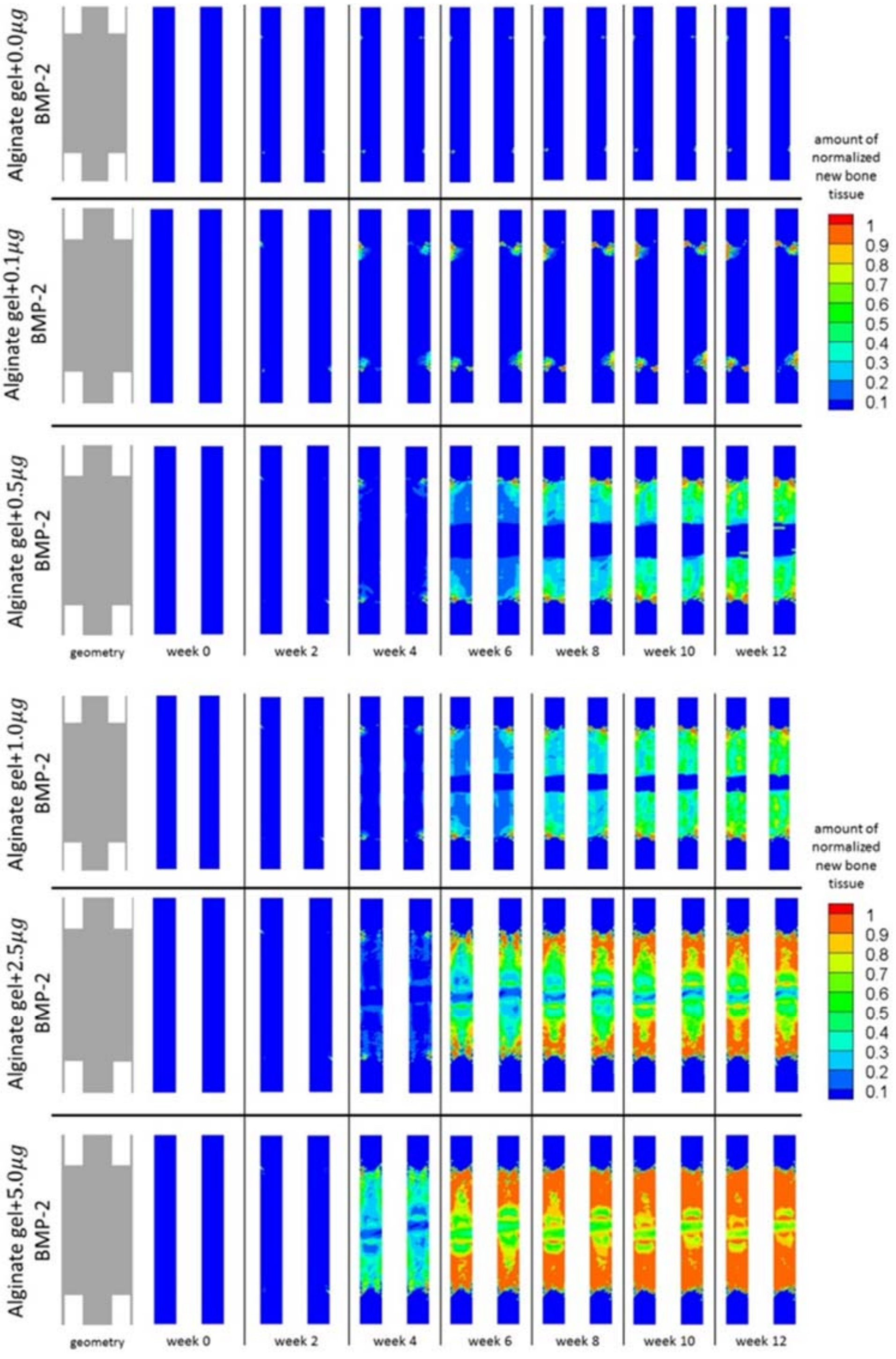

Fig. S2 – **Bone distribution for several BMP-2 doses.** Normalized bone tissue distribution for the BMP-2 doses tested ($\mathbf{0\mu g}$, $\mathbf{0.1\mu g}$, $\mathbf{0.5\mu g}$, $\mathbf{1\mu g}$, $\mathbf{2.5\mu g}$, $\mathbf{5\mu g}$) during the simulated period of 12 weeks
