# Supplementary material for: In silico Mechano-Chemical Model of Bone Healing for the Regeneration of Critical Defects: The Effect of BMP-2
Source: PLoS One. 2015 Jun 4;10(6):e0127722. doi: 10.1371/journal.pone.0127722 (PMC4456173; doi:10.1371/journal.pone.0127722)
Supplement: S3 File — (PDF) [file pone.0127722.s003.pdf]

Support Information 3

Based on the quantitative data collected from the literature, the data used can suggest different curves. Alternative modulatory curves can be tested in order to evaluate the sensitivity of the model to the variation of the main regulatory phenomenon. In Fig. S3 we propose, as suggested by the reviewer, several variations of the modulatory curves to which we shifted the peak position, peak value and asymptotic value. For each phenomenon – proliferation, hypertrophy and bone tissue production – we propose variations on the main regulatory curves as shown in Fig. S3. For the proliferation curve we propose two alternatives curves: the high peak alternative and the low peak alternative. As for the cartilage hypertrophy curve we also propose two alternative curves: the low hypertrophy alternative and the lower hypertrophy alternative. Finally, for the bone tissue production we again propose two alternative curves: the low bone tissue production alternative and the lower bone tissue production alternative. The curves in black correspond to the ones used originally hereafter referred to as reference case.

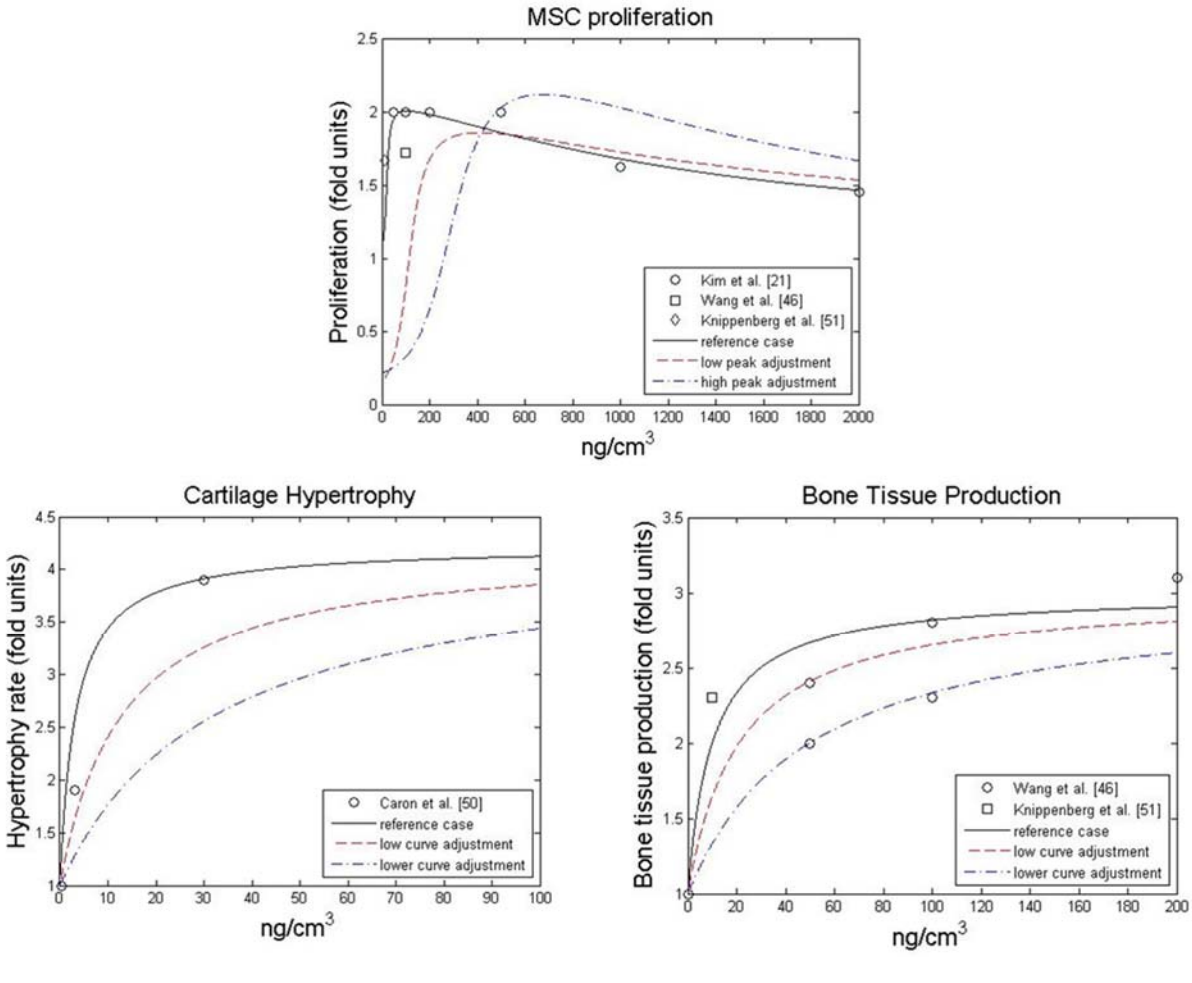

Fig. S3 - **Curves used for the sensitivity analysis of the modulatory functions.**

The results observed in Table S1, S2 and S3 suggest that the alternative curves did not cause a significant behavioral change. The reason behind such small variations has three main explanations.

First of all the mechanical stimulus in the bone defect is low given the stability provided by the external fixator. Given that we consider BMP-2 as a modulatory stimulus, if the mechanical stimulus is already low, the resulting modulated effect will also be low.

Second, one central element in our model is the hydrogel. As stated in the Methods section, the amount of hydrogel strongly controls cellular migration and therefore cellular concentration in the hydrogel. At the beginning of the simulation there are no cells inside the gel and during the simulation cells struggle to invade the hydrogel filled defect. Given that phenomena such as cell proliferation, hypertrophy and tissue production are proportional to cell population, a smaller cell population will not amplify the variations imposed by the alternative adjustments.

Finally, the alternative adjustments keep the general shape suggested by the data distribution which allows the system to conserve their behavior.

Table S1 – Bone volume deviation relative to the proposed adjustment for alternative adjustments of cells proliferation

Time (days)	High peak adj. deviation (%)	Low peak adj. deviation (%)
**0**	0,00E+00	0,00E+00
**7**	4,01E-09	5,86E-02
**14**	2,01E-03	3,34E-02
**21**	9,61E-04	2,89E-02
**28**	9,16E-04	2,52E-02
**35**	9,05E-04	2,09E-02
**42**	7,41E-05	1,54E-02
**49**	7,31E-04	1,06E-02
**56**	1,15E-03	1,28E-02
**63**	1,47E-03	1,63E-02
**70**	1,72E-03	2,08E-02
**77**	1,73E-03	2,30E-02
**84**	1,77E-03	2,34E-02

Table S2 – Bone volume deviation to the proposed adjustment for alternative adjustments of cartilage hypertrophy.

Time (days)	Low Hypertrophy ajd. deviation (%)	Lower Hypertrophy adj. deviation (%)
**0**	0,00E+00	0,00E+00
**7**	4,40E-02	4,40E-02
**14**	1,29E-02	1,29E-02
**21**	1,59E-02	1,59E-02
**28**	2,51E-02	2,51E-02
**35**	4,98E-02	4,98E-02
**42**	3,66E-02	3,66E-02
**49**	1,69E-02	1,69E-02
**56**	2,31E-02	2,31E-02
**63**	3,96E-02	3,96E-02
**70**	4,78E-02	4,78E-02
**77**	6,48E-02	6,48E-02
**84**	8,17E-02	8,17E-02

Table S3 – Bone volume deviation to the proposed adjustment for alternative adjustments of bone tissue production.

Time (days)	Low bone adj. deviation (%)	Lower bone adj deviation (%)
**0**	0,00E+00	0,00E+00
**7**	1,23E-02	3,70E-02
**14**	1,85E-02	5,79E-02
**21**	1,71E-02	2,80E-02
**28**	3,60E-02	3,89E-02
**35**	1,03E-01	1,47E-01
**42**	8,49E-02	2,70E-01
**49**	4,68E-02	1,61E-02
**56**	1,08E-01	2,84E-02
**63**	1,45E-01	5,95E-02
**70**	1,51E-01	5,34E-02
**77**	1,47E-01	5,57E-02
**84**	1,52E-01	5,08E-02
